# Effect of the COVID-19 pandemic on obesity and its risk factors: a systematic review

**DOI:** 10.1186/s12889-023-15833-2

**Published:** 2023-05-30

**Authors:** Tahir Yousuf Nour, Kerim Hakan ALTINTAŞ

**Affiliations:** 1 Department of Public Health, School of Public Health, Jigjiga University, Jigjiga, Ethiopia; 2 Department of Public Health, Faculty of Medicine, Hacettepe University, Ankara, Turkey

**Keywords:** Weight gain, Obesity, COVID-19, SARS-CoV-2, Risk factors, Systematic review

## Abstract

**Background:**

Coronavirus disease (COVID-19) is a contagious disease caused by the severe acute new coronavirus called SARS-CoV-2. Devastating social, economic, and health service utilisation-related activities. Increased burden and lifestyle changes due to confinement.

**Objective:**

This study aimed to investigate and determine the determinants of obesity during the coronavirus disease (COVID-19) pandemic from 2019 to 2023.

**Methods:**

Observational studies published between December 2019 and January 2023 were thoroughly searched using a PRISMA flow chart. PubMed, Google Scholar, Web of Science, HINARI, Scopus, and Embase databases were used. Two reviewers independently identified and critically evaluated the relevant literature. Studies that reported weight gain or involved BMI measurements of 25 kg/m2 or BMI z-scores for children during the COVID-19 lockdown were selected for inclusion. The Newcastle–Ottawa Scale (NOS) was used as a quality assessment instrument in nonrandomised studies to evaluate study quality. All the contributing determinants of weight increase were identified, gathered, and synthesised.

**Results:**

This systematic review identified 40 studies with a total population of 5,681,813 from 22 countries, of which 74.6% were male. The sample size from included articles ranged from 37 to 5,315,435. Of the 40 selected articles, 24 focused on adults, five on adolescents, three on children, and eight on children and adolescents. Physical inactivity, sedentary behaviour, bad eating habits, behavioural lifestyle, excessive stress, depression, anxiety, behavioural risk factors, sex, and ethnic minorities were associated with obesity during the COVID-19 pandemic lockdown.

**Conclusion:**

During the COVID-19 pandemic, physical inactivity, sedentary lifestyle, and poor eating patterns were the most common risk factors for obesity. Additionally, unhealthy eating habits, excessive behavioural stress, depression, anxiety, low mood, age, gender, and ethnic minorities have been identified as risk factors for obesity during the COVID-19 pandemic.

**Supplementary Information:**

The online version contains supplementary material available at 10.1186/s12889-023-15833-2.

## Introduction

In December 2019, a cluster of pneumonia cases of unknown origin was identified and linked to the Wuhan, Hubei Province, South China Seafood Market. Hospitals immediately report a disease characterised by acute respiratory distress syndrome, lymphopenia, and failure to respond to antibiotic treatment [1–3]. SARS-CoV-2 is the causative agent of COVID-19, and this brand-new coronavirus has suspected bat origins and human transmission via an unidentified intermediate host [3]. The coronavirus outbreak was formally declared an international public health emergency by the World Health Organization (WHO) on January 30, 2020 [4]. This declared emergency upended the status quo and impacted people's habits and behaviours [5].

Prior to the COVID-19 outbreak, obesity had become a global public health issue. Despite being a preventable issue, obesity has increased threefold worldwide since 1975. Universally, there are more than two billion overweight people and more than 650 million obese people, and this has become a public health issue that international organisations have labelled a pandemic even before COVID-19 [6]. Together with the pre-existing obesity problem, the COVID-19 pandemic lockdown has significantly increased the number of obese people. Worldwide, there are 650 million adults, 340 million teenagers, and 39 million obese children. The prevalence of obesity is still rising, and according to WHO future obesity projections, 167 million adults and children will experience a decline in health by the year 2025 as a result of being overweight or obese [7].

In response to the COVID-19 outbreak, authorities employed community, national, and global measures, including the lockdown of universities, schools, and public spaces. To ease the burden on the healthcare system and reduce the transmission of COVID-19, lockdown has been implemented [8]. Social confinement is considered a precaution to prevent the spread of infectious diseases from one person to another. Nevertheless, confinement has contributed to the spread of another obesity pandemic. According to several studies, lockdowns greatly influence people's eating patterns, drastically reduce their levels of physical activity, and increase the percentage of obese individuals [9, 10]. Because of the abrupt change from the normal way of life to confinement, unhealthy eating habits have occurred, which increased the prevalence of obesity during the COVID-19 pandemic [11].

These findings also raise concerns about the potential impact of the COVID-19 pandemic on long-term health, basic vaccination campaigns, nutrition services, and access to basic healthcare [12]. Before the COVID-19 pandemic, the prevalence of obesity was 11% and 15% among men and women, respectively. However, during the SARS-CoV-2 pandemic, it increased to 25.3% and 42.4% in men and women, respectively [13]. The effects of the COVID-19 pandemic on the global obesity prevalence and trends increased during the COVID-19 lockdown. Obese people are more likely to experience serious consequences from SARS-CoV-2 infection, including hospitalisation, the need for acute clinical care, and death [14–16]. To the best of our knowledge, little is known about the impact of COVID-19 on obesity and its associated risk factors. This systematic review aimed to investigate and synthesise all observational studies conducted from 2019 to 2023 on the impact of the COVID-19 on obesity and its risk factors worldwide.

## Methods

The systematic review of “effect of the COVID-19 pandemic on obesity and its risk factors: a systematic review.” was not registered on PROSPERO or any other international prospective registration databases.

### Searching strategies

Electronic literature searches were conducted between December 2019 and January 2023, and articles included in this systematic review were obtained from open-source databases such as PubMed/Medline, HINARI, Scopus, Web of Science, Google Scholar, and Embase. Additionally, all pertinent publications were manually searched for in the references of previously identified papers.

A literature search was conducted using the terms (“COVID-19”[Mesh]) OR “SARS-CoV-2”[Mesh] AND ((((“Obesity”[Mesh]) OR “Weight Gain”[Mesh]) OR “Body Mass Index”[Mesh])) OR “Overweight”[Mesh] based on PECO connecting with The Boolean operator we searched from databases mentioned above. Additionally, the study was based on the inclusion criteria. The PECO is (Population, Exposure, Comparison, and Outcome); population: all individuals irrespective of their age with weight gain during the pandemic. Exposure is a risk factor for weight gain during the COVID-19 lockdown. Comparison: Those who had weight gain and those who did not. Outcome: Increased body weight during the COVID-19 pandemic. Additional papers were found by manually examining the reference lists of all the included studies to avoid missing relevant articles.

### Study selection

This systematic review was not registered in international databases, but we followed the Preferred Systematic Review and Meta-analysis (PRISMA) guidelines and developed a systematic review [17]. Relevant articles were based on titles and abstracts. Studies on the COVID-19 pandemic during and after the lockdown on obesity were selected globally. As illustrated in Appendix I, these predefined search parameters supported a complete search strategy that used all record fields and Medical Subject Headings (MeSH) to increase the search in an advanced PubMed search and other electronic databases. Full text was extracted, read, and critically appraised. Quality was ensured and all significant factors were extracted from the included studies.

### Inclusion and exclusion criteria

#### Inclusion

Articles that fulfilled the inclusion criteria were included in this study. I) All observational studies, including cohort, case–control, and cross-sectional studies, were performed on adults, adolescents, and children. II). Anthropometric measurements with BMI assessment were reported for adults and adolescents, and children’s BMI and z-score during the pandemic, as well as reporting body weight, were considered. III). Weight was categorised as overweight if BMI was ≥ 25 kg/m^2^ and obese if BMI was ≥ 30 kg/m2 [18].

#### Exclusion

Articles that were unrelated to the topic or other species were excluded. Editorial letters, systematic reviews, non-observational study designs, full text not found, unpublished or non-peer-reviewed articles, and articles not written in English were excluded.

### Data selection

All included published articles were assessed by two authors (TY and KH), and a standardised Microsoft Excel format was used to extract all necessary information. Any differences between the authors were resolved through discussion and consensus. All relevant articles were included, and irrelevant articles were excluded. All necessary information was extracted, such as general information of the included articles, including first author, year of publication, country, study design, study setting, total sample size, sex, mean age, weight measurement, and all significant factors of the included studies. See Table 1.

**Table 1:** Systematic review included studies characteristics

s.n	Authors	Country	Study design	Sample Size	Subjects (male)	Published Year	Study Settings	BMI and (Mean ± SD)	Age (Mean ± SD)	Determinants factors	NOS score
1	Maltoni et al. [1]	Italy	P/C	51	31	2021	Facility based	BMI and waist/height Mean 2.8 (SD = 3.7)	14.7 ± 2.1 years	Increases sedentary behaviour (þ2.9 ± 2.8 h/day; *p* < 0.001); decreased physical activity (1.0 ± 1.6 h/week; p < 0.0 01); males spent more hours in sedentary behaviours (þ3.8 ± 2.7 h/day vs þ1.5 ± 2.5 h/day; p Z 0.003)	7
2	Robinson, et al. [2]	UK	CS	2002	1001	2020	Community based	BMI NA (32%)	Adult (> = 18)	lower levels of physical activity; low diet quality; diet overeating; decline in mental health; experiencing barriers to weight management	6
3	Haddad, et al. [3]	Lebanon	CS	407	198	2021	Community based	BMI Mean 0.04 (SD = 1.18) (52.1%)	Adult (> = 18)	Length of confinement in days (AOR = 1.070, 95% CI (1.034, 1.108) *p*-value = < 0.001); fear of COVID-19 AOR 0.962, 95%CI (0.927, 0.999) *P* = value = 0.046); EDE Eating concern subscale AOR 1.953, 95%CI (1.466, 2.601) *P* value = < 0.001); anxiety AOR 1.078 95% CI (1.025, 1.135);	6
4	Dasdemir, et al. [4]	Turkey	CS	395	187	2022	Hospital based	BMI/BMI Z NA (34.9%)	Mean age 15.04 ± 1.81 years	mean age of the participants was 1.81 ± 15.04 years; being obese before and during COVID-19; mean sleep quality scale scores before and during the COVID-19 (*p* < 0.01); mean Internet addiction scale scores of the participants before and during the COVID-19 (*p* < 0.01)	7
5	Arayess, et al. [5]	Netherlands	R/C	119	59	2022	Facility based	BMI z Mean was increased (+ 0.07, 0.15, 0.18)	Cased 12.6 ± (3.1) Controls 11.7 ± (2.5)	Frequency of no consultations increased (+ 0.41, *p* value = 0.025), Having a mother with obesity (+ 0.13) *p* value = 0.019)	7
6	Boukrim, et al. [6]	Morocco	CS	406	104	2021	Facility based	BMI NA (26.4%)	Adult (Mean age of 20.10 years ± 1.36)	low physical activity (AOR (95% CI, 1.9, 1.18–3.04) *p* value < 0.008); a balanced diet is protective against obesity (AOR = 0.30, [95% CI 0.15–0.61]) *p* value < = 0.001); Being male (AOR 0.243(95% CI (0.146–0.40) *p* value < = 0.0001),	5
7	Prado et al. [7]	Brazil	CS	1,828	768	2022	survey	BMI NA (50.1%)	Adult > 18 years	Both sexes were significant (*p* value < = 0.001); low Physical activity (*p* value < = 0.001)	5
8	Drieskens et al. [8]	Belgium	CS	28,029	9109	2021	Online survey	BMI NA (28.6%)	Adult > 18 years	increased their consumption of sugar-sweetened beverages (AOR = 1.39 (1.15–1.68) *p* value < 0.005) increased consumption of sugar beverages ((AOR = 1.29 (95CI, (1.04–1.60) *P* VALUE < = 0.005); increased their consumption salty or snacks (AOR = 3.6595CI (3.27–4.07) *p* value < 0.005); less physically active (AOR = 1.91 (95% CI (1.71–2.13) *p* value < 0.005); increased alcohol consumption (OR = 1.86 (1.66–2.08))	5
9	Dubnov et al. [9]	Israel	R/C	7,768	4399	2021	Facility based	weight to age- and sex, Mean was increased (0.07)	Less than 18 years	SDS all (*p* value = 0.012); Age groups of 2–5.9 (*p* value = < 0.001)	8
10	Dun, R et al. [10]	China	R/C	12 889	10,337	2021	Facility based (University)	BMI NA	17 to 27 years (M = 19, SD = 1)	COVID-19-related stress (AOR = 0.551; 95%CCI (0.254 to 0.847) *p* value = < 0.001); depression (AOR = 0.017, 95% CI (0.007 to 0.027) *p* value = 0.001)); both male and female; change in sedentary time (AOR = 0.476, 95% CI (0.460 to 0.492) *p* value = < 0.001))	7
11	Eşer Durmaz et al. [11]	Türkiye	CS	1000	208	2022	Online survey	BMI NA	18–27 years	Females spend > 2 h/day scale of effects of social media on eating behaviour (SESMEB) (*p* value < 0.01); emotional eating scale scores (*p* < 0.01); those have high score of SESMEB (rho 0.132, *p* < 0.01); moderately score of EES score (rho 0.334, *p* < 0.01); The interaction between the SESMEB and EES scores increases BMI *p* = 0.009)	6
12	Haewon Byeon [12]	South Korean	CS	50,858	26,535	2022	Online survey	BMI NA (17.9%)	12—18 years	Male (AOR 3.39, 95% CI (3.20, 3.58) *p* value < 0.001); stress perception high (AOR 1.33, 95% CI ( 1.25, 1.43) *p* value < 0.001)); stress perception moderate (AOR 1.08, 95% CI (1.01, 1.15) *p* value = 0.015); drinking soda (AOR 1.21 1.11, 1.31(*p* value < 0.001); mean sitting hours per day < 6 h (AOR 1.08, 95% CI (1.01, 1.16) *P* value 0.016))	6
13	Gülü, Yapici et al. [13]	Türkiye	CS	733	382	2022	Community based	BMI/percentile NA	0.5 year	Food addition or eating behaviour (*p* value = 0.001); Physical activities (*p* value ≤ 0.007)	6
14	He, Luo et al. [14]	China	R/C	5,963	2,976	2022	Survey	BMI/BMIz NA	10.7 ± 2.2 years	Ethnic minority, (*p* value = 0.002); older age (*p* value = < 0.001); less daily physical activity (*p* value = 0.018); reduced sleep duration ( *p* value = < 0.001); longer screen time(tv) (*p* value = < 0.001); history of COVID 19 Infection (*p* value = < 0.001)	7
15	Jayatissa, Herath et al. [15]	Sri Lanka	P/C	109	63	2020	Prospectively	HAZ/WAZ NA	26·4 (SD = 16·3) months	Household Food security status (*p* value = < 0·001)	5
16	Jia, Zhang et al. [16]	China	R/C	10,082	2,853	2021	Retrospective survey	BMI NA	19.8 ± 2.3 years	Increased average sedentary time (*p* < 0.01); the average sleeping time (*p* < 0.01); Increased screen time (*p* < 0.01); sedentary time (h/day) (*p* < 0.01); active transport for commuting/errands Housework (*p* < 0.01); activity Moderate to vigorous (*p* < 0.05); physical activity Walking for leisure (*p* < 0.01)	6
17	Jimenez, de Hollanda et al. [17]	Spain	CS	603	166	2020	Hospital based	BMI NA	18 years and above	Low mood (*p* < 0.01); dietary habits (*p* < 0.01); purchases of unhealthy food (*p* = < 0.01); snacking (*p* = 0.05); consumption of sugary beverages (*p* value = 0.02); consumption of alcohol (*p* value = 0.03)	6
18	Mai A. Khatib et al. [18]	Saudi Arabia	CS	481	184	2022	Online survey	BMI NA	18 years and above	Physical activity is protected (OR = 1.03, 95% CI (*P* = 0.008)); While increasing the quantity of meals (OR = 1.03, 95% CI (*P* = 0.009)); not adapting healthy cooking methods (OR = 1.03, 95% CI (*P* = 0.004))	7
19	Na-Hye Kim et al. [19]	South Korean	CS	147,346	83,123	2022	Survey	BMI NA	18 years and above	Level of physical activity (*p* = value 0.001); Average daily hours of sleep (*p* value = 0.001); Stress (*p* value = 0.001); Awareness of depression experience (*p* value = 0.001)	7
20	Myung-Nam Lee et al. [20]	South Korean	R/C	12,218	4311	2022	survey	BMI NA	18 years and above	Sex (AOR 2.262, 95CI (1.985, 2.577) *p* value 0.000)); age (AOR 0.617, 95% CI (0.488 0.781)) *p* value 0.000)); sitting time per day (AOR 1.023, 95%CI (1.006 1.041) *p* value 0.008)); walking time per day AOR 1.133, 95% CI (1.064 1.207) *p* value 0.000))	9
21	Miguel López-Moreno et al. [21]	Spain	CS	675	203	2020	Online survey	BMI NA	18 years and above	Age 18–36 (*p* = 0.01); sleep quality < 7 h/day (*p* = 0.01); Sex (*p* < 0.05); exercise during confinement (*p* value- 0.01); emotional eater questionnaire (*p* value < 0.001)	5
22	Serena Marchitelli et al. [22]	Italy	CC	110	32	2020	Online survey	BMI NA	Age 47.24 ± 14.3	Having stress (*p* value = 0.028); low depression for patients without a psychiatric diagnosis (*p* value = 0.019); binge eating behaviours for patients with a psychiatric diagnosis (*p* value = < 0.001)	8
23	Wudeneh Mulugeta et al. [23]	USA	R/C	11,534	7,681	2021	electronic medical records	BMI NA Female (46.1%) Male (40.6)	18 years and above	Obesity rates increased among Haitian (51.2%-55.0%, *P* < .01); Hispanic women (50.7%-51.8%, *P* < .01); 18 to 39 vs ≥ 60 years of Age (OR = 1.45, 95% CI (1.07, 1.97) *p* value < 0.005)); food and housing insecurity (OR = 1.44, 95% CI = 1.05, 1.97); tobacco use (OR = 1.38, 95% CI (1.07, 1.78) *p* value < 0.005));. among men; and 18 to 39 vs ≥ 60 years of age (OR = 1.55, 95% CI (1.25, 1.91) *p* value < 0.005)); His- panics (OR = 1.25, 95% CI (1.01, 1.54) *p* value < 0.005)); Brazilians (OR = 1.22, 95% CI (1.03, 1.45) *p* value < 0.005)); tobacco use (OR = 1.36, 95% CI (1.10, 1.69) *p* value < 0.005))	8
24	Asmaa M. Namoos et al. [24]	USA	R/C	69,510	30,904	2022	Secondary data	BMI NA	18 years and above	he African American population had a higher mean BMI (*p* value < 0.000)	7
25	Mirella Nicodemo et al. [25]	Italy	CS	100	32	2021	Online survey	BMI NA	11.8 (SD) ± 2.5) years	Feeling hungry (*p* < 0.0001); Age (*p* = 0.048); having breakfast (*p* = 0.020); Cooking (*p* = 0.006)	6
26	Hong Kyu Park et al. [26]	South Korean	CS	5,315,435	4,046,865	2022	Secondary data	BMI NA	Adolescents	Middle school boys High and middle SES (AOR 0.7, 95% CI (0.1 to 1.4) *P* value = 0.028)); decreased physical activity male (12–15 years, 50.0%– 40.5%; 16–18 years, 38.2%–34.5%; all *P* < 0.001); female Aged 12–15 years (21.9%–19.6%, *P* < 0.001); Increased sedentary time (AOR 1.0 95% CI (0.9 to 1.0) *p* value < 0.001))	5
27	Barkha P. Patel et al. [27]	Canada	R/C	115	51	2021	Secondary data	BMI/ age and sex children NA	children and adolescents	Female and males, body weight (98.29 versus 89.28 kg, (*p* < 0.001)	6
28	I Putu Suiraoka et al. [28]	Indonesia	CS	375	172	2021	School based	BMI/z score NA	Children	social factors.; lifestyle.; physical activity; followed by environmental factors	7
29	Jana Pyšná [29]	Czech Republic	CS	1456	775	2022	School based	BMI-for-age NA	mean age 12.9 year	Physical activity (*p* value = 0.034); screen (*p* value = 0.033)	6
30	Luigi Barrea et al [30]	Italy	R/C	121	43	2020	Hospital based	BMI NA	age 44.9 ± 13.3 years	daytime dysfunction (*p *< 0.001); decreased physical activity (p = 0.004); Smart working male worsening (*p* < 0.001)	6
31	Tereza Sˇ tvera ´ kova [31]	Czech Republic	CS	302	148	2021	Online survey	BMI NA	age = 10.1 ± 1.47 years	Spare time (Q1) (t (239.2) = 3.39., (*p* = 0.001)); school (Q2) ( t(236.9) = 2.97., *p* = 0.003)); Physical E (Q3) (t(164.87) = 9.85., *p* < 0.00)); recesses (Q4) (t(302) = 7.91., *p* < 0.001))	6
32	Anna Vážná et al. [32]	Czech Republic	R/C	3,518	1,759	2022	home-school based online survey	BMI NA	aged 4.71 to 17.33 year	Age trends are highly for both sexes (*p*-value < 0.001);	7
33	Paula Sol Ventura et al. [33]	Spain	R/C	3464	1727	2021	Online survey	NA	Less than 17	Gender showed that sleep (*p* value = 0.0038); Age different showed delay bedtime (*p* value < 0.0001); not adequate hours of sleep (*p* value < 0.0001); disorders of initiating and maintaining sleep (*p* value < 0.0001) Physical inactivity (OR 2.0 95% CI (1.8, 2.6) *p* value < 0.005)	6
34	Shujuan Yang et al. [34]	China	CS	10 082	2,852	2020	Retrospective survey	BMI Mean increased (21.8–22.6)	19.8 ± 2.3	Educational status increased BMI (21.3%-25.1%, *P* < .001); decreases were observed in the frequency of engaging in active transport for commuting/errands (*P* < .001); leisure-time walking during lockdown (P < .001); 34Average sedentary time increased during both workdays (*P* < .001) and weekends (*P* < .001)); well as the screen time (*P* < .001)	6
35	Qi Zhu et al. [35]	China	CS	889	347	2021	Online survey	Proportion NA	Age 31.8 ± 11.4 years	Increased food intake (*p* value < 0.001); Increased food intake for psychological factors (*p* value < 0.001); Reduced physical activity (*p* value < 0.001)	6
36	Huda Al Hourani et al. [36]	Jordan	CS	477	231	2021	Self-reported	Z-scores (BAZ, HAZ, BMI) NA	aged 6–17 years	Spent more than 3 h the screen (*p* value < 0.001); Increased physical inactivity (*p* value < 0.001)	6
37	Nassar et al. [37]	Egypt	CS	37	37	2021	survey	BMI/MBI Z NA	10.8 ± 0.46	Sleep hours per day (*p* value = .038); depression score of mothers (*p* value = .010); stress score of mothers (*p* value = .026)	8
38	Vilma K, et al. [38]	Lithuania	CS	2447	298	2020	online survey	BMI NA	Age 18 year and above	Sex being female (*p* value = 0.015); age groups of 36–50 (*p* value = 0.014); age group 18–35 is (*p* value = 0.001); Intake of carbonated or sugary drinks (*p* value = 0.049); increased Intake of fast-food (*p* value = 0.001); Increased Alcohol consumption (*p *value = 0.008); decreased Physical activity (*p* value = 0.001); snacking increased (*p* value = 0.001)	6
39	Marianna Pellegrini et al. [39]	Italy	R/C	150	34	2020	Hospital based	BMI NA	47.9 ± 16.0	Increased education (inversely, β = − 1.15; 95%CI − 2.13, − 0.17(*p* = 0.022)); Self-reported anxiety/depression (β = 1.61; 0.53,2.69 (*p* = 0.004)); not consuming healthy foods (β = 1.48; 0.19, 2.77 (*p* = 0.026))	7
40	Daniela Reyes-Olavarría [40]	Chile	CS	700	172	2020	Online survey	BMI NA	Age 18–62 years	Consumption of fried foods ≥ 3 times per week (OR 3.36,95% CI (*p* < 0.001); low water consumption (OR 1.58, 95% CI (*p* = 0.03); sedentary time ≥ 6 h/day (OR 1.85, 95% CI (*p* = 0.01)	6

Key: *P/C* Prospective cohort

*R/C* Retrospective cohort

*C/C* Case–control

*Cs* Cross-sectional

*NA* Not available

### Quality assessments

Quality rating was performed by two authors independently using the Cochrane Collaboration endorsed quality assessment tool of the Newcastle–Ottawa Scale (NOS) for non-randomised studies: a) selection, b) comparability, c) exposure for case–control, d) outcome for cohort study design with a total points of 9 and cross-sectional studies, a) selection, b) comparability, and c) exposure [19]. It was ranked based on the number of stars they achieved and classified as good quality (7–9) and satisfactory (5–6) and less than (5) was considered poor quality and excluded from the systematic review.

## Results

### Search results

**Fig. 1:**
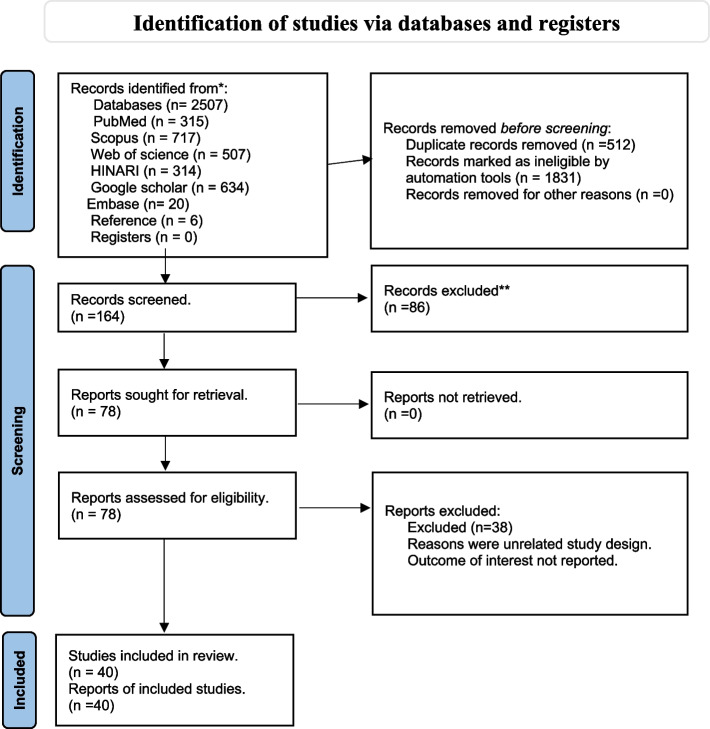
PRISMA flow diagram for the search results of selection studies

We identified 2507 articles from different electronic databases such as Pubmed/Medline, Google Scholar, Scopus, HINARI, Web of Science, and Embase. Six additional studies were manually extracted from the references of included studies. After removing duplicates using a reference manager (EndnoteX9 Thomson Reuters), 512 articles were removed. A total of 1,665 studies were screened based on the eligibility criteria. Insignificant and unrelated studies were excluded based on titles and abstracts. Patients with a non-observational study design were excluded. In total, 164 articles were screened and critically appraised based on the inclusion criteria mentioned in this study. Forty articles met the inclusion criteria of this systematic review. As depicted in Fig. 1. The quality of all the included articles was determined. It was classified as good and satisfactory in 7–9 (16 studies) and 5–6 (twenty-four studies) based on the stars provided. This number of studies was excluded based on their unsatisfactory rank of fewer than five stars**. **As shown in Fig. 1.

### Description of included articles

This systematic review showed that all included studies reported weight gain during the COVID-19 pandemic. All forty included articles were observational studies twenty-three cross-sectional, one case–control, and sixteen cohort study designs with a total participant of 5,681,813. Of the participants, 74.6% were male. The sample size ranged from 37 in Egypt [20] to 5,315,435 in South Korea [21]. The geographical distribution of the included articles was one in Belgium [22]], one in Brazil [23], one in Canada [24], one in Chile [25], five in China [26–30], three in the Czech Republic [31–33], one in Egypt [20], one in Indonesia [34], one in Israel [35], five in Italy [36–40], one in Jordan [41], one in Lebanon [42], one in Lithuania [43], one in Morocco [44], one in the Netherlands [45], one in Saudi Arabia [46], four in South Korea [21, 47–49] three in Spain [50–52], one in Sri Lanka [53], three in Turkey [54–56], one in the UK [57], and finally, two were conducted in the USA [58, 59]. Quality was assessed using the modified Newcastle–Ottawa Scale [19]. Out of included forty studies, 16 were classified as good quality [20, 26, 27, 33–38, 45, 46, 48, 49, 54, 58, 59] while twenty-four were also classified as satisfied [21–25, 28–31, 39–44, 47, 50–53, 55–57, 60]. Twenty-four articles were done on the adult population [20, 22, 23, 25, 26, 28–30, 32, 35, 36, 38, 40, 42–44, 46, 48–50, 55, 57–59], five articles were done only adolescents [31, 39, 41, 47, 60], three research articles were also done on children [34, 53, 54], and finally, eight articles were done on both children and adolescents [21, 24, 27, 33, 37, 45, 52, 56] as shown in Table 1.

### Impact of COVID-19 lockdown on body weight

To prevent contagiousness of COVID-19 spreading many countries have implemented strict quarantine law. This resulted from psychological and NCDs problems. Obesity has become a public health problem during this pandemic. The purpose of this study was to assess the impact of the pandemic on obesity. All the included studies revealed body weight changes during the pandemic. The mean weight gain during COVID-19 pandemic ranged from 0.04 (SD = 1.18) [42] to 2.8 (SD = 3.7) [37] and the prevalence of weight gain ranged from 17.9% [47] to 52.2% [42]. Despite this, some studies reported that the determinants of weight gain decreased during the COVID-19 lockdown [28, 29, 44, 46, 49].

### Factors associated with obesity during the COVID-19 lockdown

The most common determinants identified were physical inactivity [21–23, 27, 30, 31, 34, 37, 40, 43, 44, 46, 48, 52, 56, 57, 60], increasing sedentary [21, 25, 26, 28, 29, 37], and eating unhealthy foods [25, 36, 38, 43, 46, 50, 57]. In addition, the age of participants, feeling hungry, overeating, eating low-quality or unbalanced diet, sweet beverages, snacks, excess salty foods, drinking soda, binge eating, and less water consumption, we have one additional paper on household food insecurity, gender, ethnic minority, educational status, fear of COVID-19, anxiety, stress, and low mood during the pandemic, Internet or social media addiction, long screening time, long sitting hours, and substance abuse were found to be associated with COVID-19 lockdown on obesity.

### Physical inactivity

Physical inactivity was the most identified factor during the COVID-19 lockdown due to lifestyle changes during the pandemic. This finding is associated with weight gain and obesity during the quarantine period. Of the 40 studies included in this review, 17 identified physical inactivity as a risk factor for weight gain during the lockdown [21–23, 27, 30, 31, 34, 37, 40, 43, 44, 46, 48, 52, 56, 57, 60]. In contrast, five articles reported that active to vigorous physical activities decreased obesity/weight gain during pandemics [28, 29, 41, 49, 51]. As shown in Table 1.

### Sedentary behaviour and behavioural lifestyle

Sedentary behaviour notably harms human health. It is defined as low levels of energy expenditure while sitting, reclining, or lying down. Sedentary behaviour increases the risk of all-cause mortality. Six studies that assessed the increasing impact of sedentary time on weight gain during the COVID-19 pandemic [21, 25, 26, 28, 29, 37], reduced sleep hours [20, 27, 52, 54], increased Internet addiction during and after the pandemic [54], experienced barriers to weight management [57], length of confinement a day [40, 42, 60], long sitting hours [31, 47, 49, 51], tobacco use [59], unnecessary alcohol conception [22, 43], and long screen time use [27–29, 31, 41, 48] were reported as risk factors for high BMI. Another study reported female spend more time on social media [55]. A history of COVID-19 infection leads to increased body weight [27]. Finally, missing consultations were reported as a risk factor for obesity during the COVID-19 pandemic [45]. As reported in Table 1.

### Unhealthy eating behaviours

Unhealthy eating was defined as eating food that contained more calories than used, saturated fats, and food with high added sugar. Four studies identified increased consumption of sweet beverages and snacks [22, 39, 43, 50], consumption of excess salt [22], eating unhealthy foods [25, 36, 38, 43, 46, 50, 57], drinking soda [47], overeating food [30, 42, 46, 56, 57], feeling hungry [39], and eating more recesses [60]. On the other hand, household food insecurity [53, 59] and drinking less water during COVID-19 [25] were found to be significantly associated with obesity and weight gain. As depicted in Table 1.

### High level of stress, anxiety, depression

Stress affects the human brain and activates brain hormones, such as cortisol, which increase hunger and eat more foods, leading to increased body weight. In the included studies, one was for fear of COVID-19 [42], one for anxiety [42], five for increased stress [20, 38, 44, 47, 48], one for poor mood development [50], and three studies reported a statistically significant association with depression [20, 26, 48]. As shown Table 1.

## Age of participants

After the World Health Organization (WHO) announced the COVID-19 pandemic, everything changed dramatically, including individual lifestyles and social activities. Therefore, several factors responsible for obesity and weight gain have been identified. The current study identified that age which is a biological determinant was found to be one of the risk factors for obesity/ weight gain during the COVID-19 pandemic lockdown. Twelve studies identified age as a significant contributing factor to obesity/weight gain [21, 27, 33, 35, 37, 39, 43, 48, 49, 51, 52, 54]. The minimum mean age group reported ranged from mean 10.8 (SD = 0.46) [20] and the maximum mean age group reported was 47.9 (SD = 16) [36]. As shown in Table 1.

### Gender-related characteristics

Among the included genders males were dominant with 74.6%. Three studies reported that male were more likely to be obese than female [37, 44, 47], while one study claimed that females were more likely to gain weight [43], and four articles stated that both female and male were reported weight gain [23, 26, 49, 51]. As depicted in Table 1.

### Educational status and ethnicity

Three studies identified educational status as a risk factor for weight gain during the lockdown. Those educated were less likely to gain weight than those who did not have a high level of education [29, 36, 60]. The outcome of weight gain has been reported to be higher in ethnic minorities than in ethnic majority [27, 58, 59]. One study revealed that having an obese mother during lockdown was a risk factor for the child to gain weight during lockdown [45]; similarly, household food insecurity was included. As reported in Table 1.

## Discussion

This systematic review included 40 articles with 5, 681, 813 participants. COVID-19 is rapidly transmitted and has a high case fatality rate. It is associated with several short- and long-term complications. During the COVID-19 lockdown, individuals' habits dramatically changed, their calorie intake exceeded their energy calorie expenditure, and fatty tissue accumulated, which is systematically linked to other determinants such as environmental and genetic factors [61]. The COVID-19 pandemic is responsible for the re-emergence of diseases and worsening of their outcomes. The current study revealed that physical inactivity, age of participants, feelings of hunger during the lockdown, increased sedentary time, consumption of poor quality or unbalanced foods, consumption of more sweet snacks and beverages, drinking soda, binge eating, less water consumption, household food insecurity, gender, ethnic minorities, educational status, anxiety, stress and poor mood, addiction to the Internet, and social media were found to be risk factors for obesity during the pandemic. The WHO Health Organization defines physical activity as any skeletal muscle-driven motion that requires energy expenditure. Activities are performed for fun, getting to and from a destination, or for business that can be an intense and light exercise that is beneficial for individuals’ health [61].

Obesity is gradually increasing and has become a pandemic before and during the COVID-19 lockdown [62]. The current study reported that physical inactivity was a risk factor for obesity during the lockdown. This is in line with a study conducted in Italy [5], USA [63], and Poland [64]. Another study reported that poor eating, inactivity, and binge eating raise BMI [65–68]. In addition, a meta-analysis revealed that physical activity and consumption of a healthy diet lowered the risk of NCDs [69]. The possible rationale was increased bad eating habits, unhealthy food consumption, increased screen time, stress, and biological and socioeconomic risk factors responsible for high BMI during the pandemic.

During the COVID-19 pandemic, the WHO in 2020 developed a guideline recommending that both children and adolescents limit spending time on sedentary behaviours. Non-sedentary time is an interactive non-screen-based activity with a caregiver, such as reading, more active play, and getting enough good sleep. The current study showed that decreased energy expenditure, reclining, lying down, and changing patterns and quality of sleep, Internet, and social media addiction during the pandemic barrier to weight management were responsible for high BMI.

This study resonated with studies that reported longer screen time, staying up late at night, getting up late in the morning, and disturbed sleep patterns that disturbed the quality of sleep [70–73]. Similarly, increased daytime sleepiness, sleep disturbances, and physical and psychological disturbances, such as cognitive effects, led to poor performance during the COVID-19 lockdown [70, 72, 74]. Other studies revealed that Internet addiction led to restrained and long stays at home, resulting in physical inactivity, and lower sleep quality was the rationale for increased weight during the pandemic [70, 74–76].

The best strategy to prevent obesity is a healthy lifestyle, balanced diet, regular exercise, and weight reduction for at-risk groups [77]. Sedentary behaviour is a major risk factor for obesity. A lengthy stay at home. Unhealthy or junk food with elevated levels of fat, salt, and sugar, as well as foods lacking essential nutrients such as fibre, vitamins, and minerals, are responsible for both short- and long-term health outcomes. The pandemic reversed normal lifestyles and facilitated easy access to unhealthy food and unhealthy behaviors. This systematic review reported that unhealthy eating, high-calorie intake, intake of saturated fats, snacks, high-added sugar, sweet beverages, consumption of excess salt, and drinking of soda were risk factors identified during the COVID-19 lockdown. Studies done in different parts of the world showed that unhealthy diets, sweetened snacks, and salty food were risk factors for increased BMI [78, 79]. Unhealthy foods and physical inactivity have been reported to be risk factors for obesity [80, 81]. Evidence has shown that negative emotional eating, associated with physical inactivity and sleep duration, leads to increased weight [82]. Unhealthy eating habits and increased consumption of snacks and food after dinner are related to obesity [83]. In contrast, eating a healthy diet, performing social activities, and decreasing post-dinner food during the pandemic [64, 84] and consumption of a Mediterranean diet decreases body weight [83]. A probable reason for this is that physical inactivity, unhealthy food consumption, and sedentary time increased the BMI during the COVID-19 pandemic. This study identified that psychological problems during COVID-19 were responsible for the high BMI. Elevated levels of stress, depression, anxiety, and low mood during the COVID-19 lockdown were recognised as the consequences of increased BMI. Our findings are in line with those of another study conducted in the UK [85]. University-based studies in Saudi Arabia during the COVID-19 quarantine increased psychological manifestations, which increased the levels of depression, anxiety, and stress [86]. Similarly, a high prevalence of depression and anxiety during the pandemic resulted in unhealthy eating behaviour at night and stress eating, which lasted with increased body weight [87, 88].

Increased serotonergic neuronal activity and tryptophan levels in the body aggravate excessive consumption of carbohydrate-rich foods and less protein-rich meals, leading to increased body weight [89]. This physiological change affects well-being, triggering a wide range of psychological problems such as panic disorder, anxiety, and depression during the COVID-19 pandemic. This systematic review found that sex was a risk factor for high BMI during the COVID-19 pandemic. Being male [37, 44, 47], female [90], or both were responsible for the increased weight during the lockdown. Several studies have found males more likely to be obese than females [91, 92]. Another study reported that females gained less weight than male participants [93]. In contrary females were more likely to develop a high BMI during the pandemic [43]. Four studies reported increases in BMI, regardless of gender BMI increases [23, 26, 49, 51]. One explanation may be that the studies were conducted in different areas, sample sizes, and methodologies, which may have resulted in different results. During the pandemic, men commonly perform indoor exercises.

Educational status plays a significant role in behavioural changes and the immediate adoption of modifiable lifestyles. The present study highlighted that higher educational status decreases BMI due to the identification and management of risk factors. A similar study conducted in the USA found that the educational level of college graduates may overcome weight gain and obesity risks [94]. It is possible that they were early acceptors and implementors because they could search for resources without help. Another study revealed that increased knowledge of nutritional status is responsible for manging weight gaining [95]. Low-educated individuals are late acceptors because of their cultural background, and they are sometimes fragile in accepting misinformation, disinformation, and rumours.

The current systematic review found that ethnic minority groups were an element of an increased BMI. A minority group refers to a group of people whose practices, race, religion, ethnicity, or other characteristics are fewer than the main groups of the classifications or communities that live with [96]. Ethnic minorities are the most disadvantaged and poorer individuals, and most of them were affected by the COVID-19 lockdown. During the COVID-19 pandemic, they suffered from inequality, including infection-related issues [97]. Mortality during the pandemic has also increased for minority groups [98] and access to health facilities such as hospitalisation [99].

A more common reason for the socioeconomic status (SES) gradient in health is that typically poor people make unhealthy lifestyle choices. that was responsible for several types of NCDs, including obesity, before and during the pandemic. A recent systematic review showed that ethnic minorities are a risk factor for obesity during the pandemic lockdown [27, 58, 59]. This is in line with several studies showing that SES is also a risk factor for obesity during the COVID-19 lockdown [28, 100]. A possible reason for this is the socio-economic factors that result in not accessing health services easily due to financial constraints [101, 102]. Minority groups are mostly affected by infectious diseases because of stigma and discrimination, which simultaneously affect NCDs, injuries, and mental illnesses. The current study reported that food insecurity was a risk factor for weight gain during the COVID-19 lockdown. Similar studies have identified [103] a possible reason for this irregular dietary pattern [104].

Lastly The cellular mechanisms associated with obesity and COVID-19 are not yet fully understood. however, current studies have provided evidence of various pathways that may contribute to the increased risk of severe COVID-19 in individuals with obesity. This may be because the angiotensin-converting enzyme 2 (ACE2) receptor is elevated in obese individuals, increasing their susceptibility to COVID-19 [105]. The ACE2 receptor has been identified as the primary entry point for SARS-CoV-2 [106]. Adipose macrophages produce cytokines such as interleukin-6 (IL-6) and tumour necrosis factor-alpha (TNF-α) which exacerbate the inflammatory response to SARS-CoV-2 infection and lead to severe COVID-19 in individuals with obesity [107]. Moreover, severe cases of COVID-19 have been linked to a phenomenon known as cytokine storm, which is characterised by the excessive production of pro-inflammatory cytokines [108, 109]. This cytokine storm may be more likely to occur in obese individuals because of their chronic inflammatory state and dysregulated immune response [110].

### Limitation and strength

The exclusion criterion is one of the limitations of this study. Most of the included studies were facility-based, which is unsuitable for generalisation. Only studies conducted in English were included, and other languages were excluded. Second, because most of the included studies used secondary data, there was a chance of incomplete data. Third, most of the included studies were cross-sectional studies that were unable to determine the temporal frame between exposure and outcome. Fourth, the bulk of studies conducted online utilised self-report of data, and participants may have provided information that may have been slanted in their favour about the quality of diet, and measurement systematic error may have been present.

The strength of the current systematic review is that studies with large sample sizes were included, and most of the included studies used an instruction manual to provide accurate data. Through this systematic review, we were able to identify the risk variables for being overweight or obese, which will guide future studies.

## Conclusion

The current study concluded that sociodemographic factors, physical inactivity, sedentary lifestyles, reduced sleep quality, increased technology utilization, harmful substance abuse, unhealthy food consumption, and psychological problems were the most common obesity risk factors during the COVID-19.

### Recommendation

Comprehensive assessment of risk factors is required to reduce the effects of NCDs. NCD prevention should be implemented at four levels. Global, national, societal, and individual levels. The individual level is simple to complete. Modifiable risk factors are addressed through health promotion, and primary and secondary prevention. The use of WHO guidelines for healthy eating, physical activity, preconception and prenatal care, early childhood diet and activity, healthy nutrition and activity for older children, and weight management should be promoted. Future research should identify the risk factors that contribute to weight gain and build on existing strategies. Qualitative research on behavioural and psychological obesity risk factors is recommended.

## Supplementary Information

**Additional file 1: Supplementary Table 1.** Quality assessment checklist for included cohort studies.

**Additional file 2: Supplementary Table 2.** Quality assessment checklist for included case-control studies.

**Additional file 3: Supplementary Table 3.** Quality assessment checklist for included cross-sectional studies.

## Data Availability

All data generated or analysed during this study are included in Supplementary Table 1.

## Abbreviations

BMI: Body Mass Index
LMIC: Low- and Middle-Income Countries
MeSH: Medical Subject Headings
NOC: Newcastle-Ottawa Scale
PRISMA: Preferred Reporting Items of Systematic Reviews and Meta-Analysis
WHO: World Health Organization
SESMEB: Scale of Effects of social media on Eating Behaviour
EES: Emotional Eating Scale
SDS: Specific Deviation Score
CI: Confidence Interval
AOR: Adjected Odd Ratio
