# Correction to: Effect of the COVID‑19 pandemic on obesity and its risk factors: a systematic review

**DOI:** 10.1186/s12889-025-21888-0

**Published:** 2025-03-17

**Authors:** Tahir Yousuf Nour, Kerim Hakan ALTINTAŞ

**Affiliations:** 1 Department of Public Health, School of Public Health, Jigjiga University, Jigjiga, Ethiopia; 2 Department of Public Health, Faculty of Medicine, Hacettepe University, Ankara, Turkey

**Correction to: BMC Public Health 23, 1018 (2023)**

**https://doi.org/10.1186/s12889-023-15833-2**

The original publication of this article [1] contained the following errors:

References 2, 3 and 4 were incorrect

Several typographical errors

The incorrect and correct information is shown in this correction article. The original article has been updated. This does not affect the results nor conclusions of the article.

**Table:** 

Incorrect	Correct
This systematic review aimed to investigate and synthesise all observational studies conducted from 2019 to 2023 on the impact of the COVID-19 **lockdown** on obesity and its risk factors worldwide.	This systematic review aimed to investigate and synthesise all observational studies conducted from 2019 to 2023 on the impact of the COVID-19 on obesity and its risk factors worldwide.
Out of included forty studies, 16 were classified as good quality [26, 27, 33,34,35,36,37,38, 45, 46, 48, 49, 54, 58, 59] ….	Out of included forty studies, 16 were classified as good quality [20, 26, 27, 33,34,35,36,37,38, 45, 46, 48, 49, 54, 58, 59]
Twenty-four articles were done on the adult population [22, 23, 25, 26, 28,29,30, 36, 38, 42,43,44, 46, 48,49,50, 55, 57,58,59]….	Twenty-four articles were done on the adult population [20, 22, 23, 25, 26, 28,29,30, 32, 35 36, 38, 40, 42,43,44, 46, 48,49,50, 55, 57,58,59]
Figure 1: N = 1025	Figure 1: N = 717
The purpose of this study was to assess the impact of the pandemic lockdown on obesity.	The purpose of this study was to assess the impact of the pandemic on obesity.
….four studies [20, 38, 42, 44, 47, 48, 50] reported a statistically significant association with depression [20, 26, 48]. As shown Table 1.	….three studies reported a statistically significant association with depression [20, 26, 48]. As shown Table 1.
Among the included male were dominant with 74.6% were male.	Among the included genders males were dominant with 74.6%.
The outcome of weight gain has been reported to be higher in ethnic minorities than in ethnic minorities [27, 58, 59].	The outcome of weight gain has been reported to be higher in ethnic minorities than in ethnic majority [27, 58, 59].
The COVID-19 pandemic is responsible for the re-emergence of chronic diseases and worsening of their outcomes.	The COVID-19 pandemic is responsible for the re-emergence of diseases and worsening of their outcomes.
The pandemic reversed normal lifestyles and facilitated easy access to unhealthy food and healthy behaviours.	The pandemic reversed normal lifestyles and facilitated easy access to unhealthy food and unhealthy behaviours.

Incorrect References 2-4

2. Rundle AG, et al. COVID-19 related school closings and risk of weight gain among children. Obesity (Silver Spring, Md). 2020;28(6):1008.

3. Xiang M, Zhang Z, Kuwahara K. Impact of COVID-19 pandemic on children and adolescents’ lifestyle behavior larger than expected. Prog Cardiovasc Dis. 2020;63(4):531.

4. Guo Y-R, et al. The origin, transmission and clinical therapies on coronavirus disease 2019 (COVID-19) outbreak–an update on the status. Mil Med Res. 2020;7(1):1–10.

Correct references 2-4

2. WHO. *Pneumonia of unknown cause – China*. 2020 [cited 2023 22 February 2023]; Available from: https://www.who.int/emergencies/disease-outbreak-news/item/2020-DON229.

3. Zhou, P., Yang, X.L., Wang, X.G., Hu, B., Zhang, L., Zhang, W., et al. (2020) A Pneumonia Outbreak Associated with a New Coronavirus of Probable Bat Origin. Nature, 579, 270-273. https://doi.org/10.1038/s41586-020-2012-7

4. WHO. C*OVID-19 Public Health Emergency of International Concern (PHEIC) Global research and innovation forum*. 2020 [cited 2023 2023]; Available from: https://www.who.int/publications/m/item/covid-19-public-health-emergency-of-international-concern-(pheic)-global-research-and-innovation-forum.
